# Supplementary material for: Estimated true prevalence and associated risk factors of bovine paratuberculosis antibodies in dairy herds in Bangladesh
Source: PLoS One. 2026 Jan 22;21(1):e0334683. doi: 10.1371/journal.pone.0334683 (PMC12826488; doi:10.1371/journal.pone.0334683)
Supplement: Attachment — Submitted filename: Response to Reviewers.docx [file pone.0334683.s007.docx]

We are grateful for the valuable comments and suggestions from the academic editor and both reviewers, which have resulted in an enhanced manuscript. The modifications we implemented based on their feedback are indicated as tracked changes in the revised manuscript.

**Academic Editor’s Summary**

The paper has now been reviewed by an expert in the field. While the reviewers acknowledge that your study addresses an important gap — namely the absence of data on Mycobacterium avium subsp. paratuberculosis (MAP) in Bangladeshi dairy herds — they have identified several issues that must be resolved before the manuscript can be considered further. After careful consideration of the reviewer’s comments and my own evaluation, I am returning the manuscript for major revision. The reviewer appreciates the novelty and potential value of your work but notes that the current version overstates its representativeness and certainty. Specifically, the survey’s non-random sampling, limited geographical balance, and use of milk ELISA only restrict the generalizability of the conclusions. Furthermore, several methodological details and interpretations require clarification or revision.

Response: We have thoroughly addressed all possible reviewer and editorial concerns. The revised manuscript now clearly acknowledges the study’s limitations, such as the non-random sampling design, limited geographic representation, and the diagnostic scope of the milk ELISA, with appropriate caution in interpretation and generalizability. Methodological transparency has been improved by including detailed descriptions of the sampling rationale, herd selection criteria, laboratory procedures, and statistical analyses to ensure clarity and reproducibility. Interpretations and conclusions have been refined to moderate any overstatements of representativeness or certainty, ensuring that all claims are fully supported by the study design and data. Additionally, minor editorial and structural revisions were made throughout the manuscript to enhance overall readability, coherence, and scientific precision.

**Reviewer #1**

**General Assessment**

- The study is valuable but needs clarification and moderation in claims.
- Representativeness and diagnostic certainty are overstated.
- The term “True prevalence” in the title may mislead readers.

**Detailed Comments**

**Title and Abstract**

- The phrase “True prevalence” could mislead readers to assume diagnostic certainty; consider “Estimated true prevalence.”

Response: We have revised the term ‘true prevalence’ to ‘estimated true prevalence,’ following the reviewer’s recommendation..

- The abstract contains excessive numerical detail i.e. confidence intervals and regression coefficients, that might be summarized more succinctly for readability.

Response: Excessive numerical details were summarized in the abstract.

- The term “nationwide” might be overstated given sampling in only 14 districts.

Response: We have deleted the term ‘nationwide’ from abstract.

**Introduction**

This section provides a comprehensive background on Mycobacterium avium subsp. paratuberculosis (MAP), its global distribution, and regional context and clearly identifies the knowledge gap and study rationale for Bangladesh.

**Materials and Methods**

- The sampling strategy for herd selection is described as “convenient,” which introduces potential bias and limits national representativeness.

Response: We agree with the reviewer that the purposive sampling strategy introduces potential bias and limits national representativeness. This limitation is now explicitly acknowledged in the Discussion, and Abstract, and we have clarified that the findings apply only to the study population and should not be interpreted as nationally representative.

- The criteria for selecting 14 districts and seropositive herds for individual sampling are not fully explained.

Response: The criteria for selecting 14 districts and testing positive herds for individual sampling are described in the revised manuscript.

- The diagnostic sensitivity and specificity values for the ELISA (herd-level Se = 9.58%) seem unusually low; justification or source verification is necessary.

Response: We have used the appropriate sensitivity (Se) and specificity (SP) of the ELISA utilized in this study and have reanalyzed the data.

**Results**

- Results are systematically presented from descriptive statistics to modeling outcomes. Figures and tables are well structured and appropriately referenced. The clear distinction between apparent and true prevalence is valuable.
- The jump from apparent to true herd-level prevalence (7.2 % → 71.2 %) seems implausibly large; discussion of possible model over-correction or low-test sensitivity should appear here.

Response: We have reanalyzed the data using new and suitable prior information and have provided a revised description of the true estimated prevalence in the manuscript..

- Map need better captions specifying sample numbers per district.

Response: We thank the reviewer for pointing this out. The figure caption has been revised to clarify that n refers to the number of herds sampled in each district, rather than the number of individual animals.

**Discussion**

This section is well written and compares results effectively with studies from South Asia and other countries.

Also appropriately discusses diagnostic limitations and implications for disease control.

Response: We thank the reviewer for this suggestion. In response, we have revised the discussion to explicitly address the diagnostic limitations of ELISA, including its imperfect sensitivity and specificity, which can lead to underestimation of true herd-level MAP prevalence. We have also highlighted the implications for disease control, emphasizing that accurate prevalence estimation through standardized protocols and adjusted or complementary testing is essential for effective surveillance and intervention.

Integrates productivity and reproductive performance findings logically.

Response: We thank the reviewer for this suggestion. The discussion has been revised to better integrate findings on productivity and reproductive performance. We now emphasize that MAP seropositivity is associated with modest reductions in milk yield as well as increased odds of reproductive disorders, and we discuss these outcomes in a biologically plausible context. Additionally, we clarify that these findings represent associations rather than causal relationship.

**Reviewer #2 Comments**

**General Assessment**

The manuscript describes the outcome of a survey to evaluate the prevalence of paratb in milk samples in Bangladesh. Although the manuscript contains useful information, as nothing was known about the prevalence in the county before, it has some weaknesses which should be addressed before it can be considered for publication. The main issues are, that the survey was not statistically balanced and therefore does not reflect the situation in the whole country. Furthermore, the authors tend to overinterpret their results and the manuscript lacks a critically discussion, addressing the limitations of the study. Specific comments are provided below which should be addressed during a thorough review of the manuscript.

**Specific Comments (by line number)**

**Abstract & Introduction**

**Line 22:** Why seroprevalence? Serum was not tested throughout the study.

Response: We appreciate the reviewer’s careful observation. The term *seroprevalence* has been replaced with *antibody prevalence* throughout the manuscript to accurately reflect that milk, rather than serum, was tested for antibodies against *Mycobacterium avium* subsp. *paratuberculosis*.

**Line 32-35:** There were some more significant risk factors, please list them all

Response: We thank the reviewer for the comment. Only variables significant in the final multivariable models are reported as key results (Table 2 for herd-level, Table 4 for cow-level). Variables associated in univariable screening are part of the model-building process and are listed in Table 1 and Table 3 for transparency but are not described as final findings.

**Line 63:** „…limited sensitivity in early stages of the infection, meaning…”

Response: Thank you for your edit. We have made the suggested modifications.
**Line 66:** “…the paratuberculosis status and prevalence…”

Response: Thank you for your edit; we have made the suggested modifications..

**Line 70: “…PCR (polymerase chain reaction).”**

Response: Elabortaed as suggested.
**Line 71:** “…ELISA (enzyme linked immunosorbent assay) …”

Response: Elabortaed as suggested.

**Line 71:** specify “other tests”

Response: Other test was specified.
**Line 71:** Why approximately? Be precise

Response: We have deleted the word approximately.

**Line 72:** Replace “although” by “and” or “while”

Response: We removed that portion of the sentence.
**Line 73:** Please specify large, how many animals?

Response: We have rephrased the sentence.

**Line 77:** What kind of skin test?

Response: We have replaced ‘skin test’ with the delayed-type hypersensitivity test.

**Line 96:** Please rephrase, as farms >25 lactating cows still are small and should not be addressed as large-scale herds, although this might be the case for the situation in Bangladesh.

Response: We have revised the statement based on the reviewer’s suggestion..
**Line:** Why was the expected prevalence 50%? Above it is stated that it is around 30-40% in other Asian countries. Please justify.

Response: Although published studies from parts of Asia report MAP seroprevalence around 30–40%, we used an expected prevalence of 50% in the sample-size formula because this value maximizes the binomial variance $p(1-p)$ and therefore yields the largest (most conservative) sample size for a given precision. Using the same precision (d = 0.035), the required sample size would have been 784 animals with $p=0.50$, whereas using a more realistic estimate of 35% would have produced a smaller requirement of approximately 715 animals. Because we ultimately collected 880 samples, our achieved sample size exceeds both estimates, ensuring greater statistical power, narrower confidence intervals, and sufficient coverage for subgroup analyses. Thus, the final sample size remains more than adequate regardless of whether 35% or 50% is used as the expected prevalence.

**Line 108:** How were the farms selected for the convenient sampling?

Response: The herd selection strategy has been described in the revised manuscript.

**Line 109:** If bulk milk samples were tested, why “seropositive” herds? Were there serum samples taken also? Please check and change throughout the manuscript.

Response: We have changed seropositive to LEISA positive herds.
**Line 110:** Provide more details of the farms and cows enrolled in the study, move lines 200-213 to this part of the manuscript, as they are a description of the study population and not a result of your study.

Response: The farms and cow enrollment have been described in detail.
**Line 110:** Provide manufacturer for the software

Response: Manufacturer detail provided
**Line 113:** Each cow of the farm or from selected cows? Was this a combined ample of all for quarters?

Response: We thank the reviewer for this comment. We confirm that milk samples were collected from each individual cow, not from a subset of animals. For each cow, a composite sample was taken by pooling milk from all four quarters. The sentence in the Methods section has been revised accordingly.

**Line 115:** Department for Medicine of which institution?

Response: The Institute of the department added in the revised manuscript.

**Line 117:** What are pretested questionnaires? How were they pretested?

Response: We appreciate the reviewer’s query. By “pretested questionnaires,” we refer to structured questionnaires that were piloted before the main survey to ensure clarity, relevance, and consistency. The questionnaires were tested on a small number of farms and animals outside the study population. Based on feedback from farmers and field staff, several questions were refined to improve wording and reduce ambiguity. This has now been clarified in the revised Methods section.

**Line 119:** Purchase/trading of animas and contact to other herds (pasture etc.) are well known risk factors for the transmission of MAP. Why were they mot included in the survey?

Response: Thank you for this valuable comment. All enrolled herds practiced zero-grazing and were managed entirely indoors, eliminating inter-herd contact through pasture. Furthermore, reliable records on animal purchase or trading were not available. For these reasons, these variables could not be included in the survey. This clarification has been added to the revised manuscript.

**Line 137:** (ODNC)

Response: Thank you, corrected.
**Line 150:** Where were the cut off values taken from? Manufacturer?

Response: Yes, the cutoff value was taken from the manufacturer.

**Line 163-164:** Are these the Sp and Se values for milk? Please provide a reference, the values could not be found on the home page of the producer of the ELISA.

Response: Thank you for your comment. During the initial submission, we could not find published herd- or cow-level sensitivity (Se) and specificity (Sp) values for the iELISA used to detect antibodies in bulk or individual cow milk. Although we contacted the manufacturer, the values provided were unpublished. Following your suggestion, we identified two published studies reporting these diagnostic performance estimates: for herd-level prevalence, we used an iELISA sensitivity of 63% (95% CI: 41–81%) and specificity of 92% (95% CI: 86–96%) [Reference 20]; for cow-level prevalence, we used a commercial ELISA sensitivity of 85.7% (95% CI: 76.4–95.1%) and specificity of 95.1% (95% CI: 92.6–97.7%) [Reference 21]. Using this prior information, we have updated and redone the estimation of true antibody prevalence.

**Line 202:** Delete .0 for the mean of 119

Response: Deleted as suggested

**Line 209:** How were the sampled cows distributed to the farms? In the end, only 4 farms were included concerning single animal testing in the whole survey, although you identified 10 positive farms.

Response: Thank you for your comment. To clarify, ten herds were initially identified as ELISA-positive. From these, 880 cows were randomly selected using the ‘RANDBETWEEN’ function in Excel to ensure unbiased selection. However, the inclusion of herds for individual cow-level testing was limited by herd owner willingness and logistical feasibility, resulting in only four herds contributing cows for individual sampling. Both herd-level bulk milk testing and cow-level individual testing were used in this study, and we have revised the manuscript to clearly explain the distribution of sampled cows across herds.

**Line 211:** please check your numbers, this is not possible: If there was a cow with 0 calves included, the lactation period can not range from 1-11 months.

Response: We thank the reviewer for pointing this out. Upon careful review, we identified a typographical error in the number of calves recorded for one cow. This error led to an incorrect representation of the lactation period range. The data have now been corrected, and the summary statistics have been updated accordingly.
**Line 212:** How was the body weight determined?

Response: We appreciate the reviewer’s query. The body weights reported in our study were obtained from the herd records. Herds use either digital weighing scales or other standard on-farm methods to measure the body weight of cows, and these measurements were recorded as part of their routine management practices.

**Line 221:** Seroprevalence/Milk?

Response: All instances of “seroprevalence” in the manuscript have been updated to “antibody prevalence” in the revised version to accurately reflect the measured outcome.
**Line 222:** In line 107-109 it is stated, that 10 out of 138 sampled herds were positive = 13.8%, here a prevalence of 7.2% is calculated, how? And why seroprevalence again? Was serum sampled or only milk?

Response: The calculation of herd-level prevalence is correct: 10 positive herds out of 138 sampled corresponds to 7.2% herd-level prevalence. Additionally, in the revised manuscript, all instances of “seroprevalence” have been replaced with “antibody prevalence” to accurately reflect that only milk samples were analyzed, not serum.
**Line 228:** Seroprevalence?

Response: Corrected as per the previous comment.

**Line 230:** Seroprevalence/Milk?

Response: Corrected as per the previous comment.
**Fig. 3:** What does the line in the middle of the figures stand for? Add key

Response: The middle vertical line, labeled as the mean, has been added to the figure caption.
**Fig. 5:** Add key (SP), each figure hast to be self-explaining without connection to the text of the manuscript.

Response: SP represents the sample-to-positive ratio (SP%), as now clarified in the figure caption.

**Line 267-269:** This paragraph should be moved to the beginning of the results, as these are quite general outcomes of the study

Response: Moved accordingly.
**Table 1:** The variable “semen source” is not explained at any point of the manuscript. What is source 1, 2 etc.?

Response: The variable “semen source” refers to different artificial insemination (AI) providers, coded as 1, 2, etc., to maintain confidentiality. The actual herd or AI provider names have not been disclosed due to sensitivity considerations within the AI industry.

**Table 1 and 2:** Add key and level of significance

Response: We thank the reviewer for the comment. The level of significance is already indicated by the P-values provided in the tables.
**Line 310:** This gap was not reveled by the study, this is obviously the case when diagnostic tests have a low sensitivity, delete statement.

Response: Deleted.

**Line 314-320:** Please be more modest when interpreting the results of your study. The survey was not statistically balanced for the whole country, milk samples were used only (which are known not to be the best medium for Paratb detection), the herds showed a high variety in size. All this should be considered and discussed critically. Fig. 1 for example shows, that the herd prevalence was 0% in one province and 100% in a neighbor province-this does most probably not reflect the real situation but is caused by the study design.

Response: We acknowledge that the survey was not fully representative, that herd sizes varied, and that milk samples—suboptimal for MAP detection—were used. The extreme herd-level prevalence shown in Fig. 1 likely reflects these study limitations rather than true regional differences. We have revised the discussion to highlight these points and advise cautious interpretation of the results.

**Line 321-326:** Same as above, consider and discuss weaknesses of the study and interpret with caution.

Response: Limitation of the study added.
**Line 339:** There are also some studies showing, that cows with a high milk yield more often are MAP-positive, this also should be discussed.

Response: We agree that the relationship between MAP infection status and milk yield is complex and that some studies have reported higher milk production among MAP-positive cows. In our study, we evaluated only the univariable association between S/P% values and milk yield. Because S/P% reflects antibody levels rather than confirmed MAP infection, the observed negative association should not be interpreted as a direct effect of MAP infection on milk production. We have now revised the Discussion to clarify these points and to acknowledge the conflicting evidence in the literature.

**Line 344-349:** This should be discussed to a further extend, only because there is an association between two findings it doesn’t necessary imply that they are related. There is much more literature available concerning risk factors for Paratb in dairy cows. Also, some well known risk factors (purchase, contact to other herd) were no included in this study. Also, some found strong associations like age are not discussed at all.

Response. We agree that an observed association does not necessarily imply causation. In our study, some well-known risk factors such as animal purchase or contact with other herds could not be evaluated because all participating herds practiced zero-grazing and were managed exclusively indoors. Additionally, reliable records on animal purchase or trading were unavailable. Consequently, these variables were not included in the analysis. We have now clarified this in the revised manuscript and emphasized that the associations we report—such as with age—reflect correlations within the specific management context of these herds rather than definitive causal relationships.

**Line 325-355:** Not really a result of the study, delete. On the other hand, the main results (prevalence on the herd and individual level) should be added to the conclusions.

Response: The conclusion section has been completely rewritten.

**Additional Journal / Editor Requirements**

1. **Formatting:**
   Follow PLOS ONE style templates for manuscript and title page.
   - [Main body template](https://journals.plos.org/plosone/s/file?id=wjVg/PLOSOne_formatting_sample_main_body.pdf)
   - [Title/authors template](https://journals.plos.org/plosone/s/file?id=ba62/PLOSOne_formatting_sample_title_authors_affiliations.pdf)

Response: PLOS ONE style followed

2. **Permits:**
   Add details of field/site access permits or state that no permits were required (and why).

Response: Not applicable

3. **Funding Statement:**
   Add: *“The funders had no role in study design, data collection and analysis, decision to publish, or preparation of the manuscript.”*
   Remove funding text from acknowledgments.

Response: The following funder role was added: “The funders had no role in the study design, data collection and analysis, decision to publish, or preparation of the manuscript.” Additionally, the funding text was removed from the acknowledgments section.

4. **Ethics Statement:**
   Must appear **only in the Methods section**.

Response: The ethics statement has been relocated to the methods section.

5. **Figure 1 (Map Issue):**
   Cannot use copyrighted map (e.g., Google Maps).
   Replace with a CC BY 4.0–compatible map (e.g., Natural Earth, CIA maps, or QGIS-generated).

Response: Figure 1, generated using QGIS software, does not include any copyrighted materials.

6. **Supporting Information Files:**
   The R Markdown files could not be opened — revise and re-upload.

Response: The .Rmd files were replaced with .txt files.
